# Causal mediation analysis: what is it and how can it be used to inform practice and policy?

**DOI:** 10.1093/fampra/cmaf043

**Published:** 2025-06-24

**Authors:** Pamela Fernainy, Claire Godard-Sebillotte, Anais Lacasse, Géraldine Layani, Cristina Longo, Janusz Kaczorowski, Maria Alejandra Rodriguez, Marie-Eve Poitras, Mylaine Breton, Marie-Thérèse Lussier, Yves Couturier, Catherine Hudon, Nadia Sourial

**Affiliations:** Department of Health Management Evaluation and Policy, School of Public Health, Université de Montréal, 7101 Park Avenue, Montreal, Quebec H3N 1X9, Canada; Research Centre of the Centre Hospitalier de l’Université de Montréal (CRCHUM), 900 rue Saint‑Denis, Montreal, Quebec H2X 0A9, Canada; Faculty of Medicine and Health Sciences, Division of Geriatrics, Department of Medicine, McGill University, Jewish General Hospital, Room E‑0012, 3755 Côte‑St‑Catherine Road, Montreal, Quebec H3T 1E2, Canada; Research Institute of the McGill University Health Centre, 2155 Guy Street, Suite 500, Montreal, Quebec H3H 2R9, Canada; Department of Health Sciences, University of Quebec in Abitibi-Témiscamingue, 445 boulevard de l’Université, Rouyn‑Noranda, Quebec J9X 5E4, Canada; Research Centre of the Centre Hospitalier de l’Université de Montréal (CRCHUM), 900 rue Saint‑Denis, Montreal, Quebec H2X 0A9, Canada; Department of Family and Emergency Medicine, Université de Montréal, 2900, boulevard Édouard-Montpetit, Montreal, Quebec H3T 1J4, Canada; Faculty of Pharmacy, Université de Montréal, Pavillon Jean-Coutu, 2940 chemin de Polytechnique, Montreal, Quebec H3T 1J4, Canada; Research Centre of the Centre Hospitalier de l’Université de Montréal (CRCHUM), 900 rue Saint‑Denis, Montreal, Quebec H2X 0A9, Canada; Department of Family and Emergency Medicine, Université de Montréal, 2900, boulevard Édouard-Montpetit, Montreal, Quebec H3T 1J4, Canada; Department of Family Medicine, McGill University, 5858 chemin de la Côte‑des‑Neiges, Suite 300, Montreal, Quebec H3S 1Z1, Canada; Department of Family Medicine, Université de Sherbrooke, 3001, 12e Avenue Nord, Sherbrooke, Quebec, J1H 5N4, Canada; Faculty of Medicine and Health Sciences, Department of Community Health Services, Université de Sherbrooke, 250, rue de la Faculté, Sherbrooke, Quebec J1K 2R1, Canada; Department of Family and Emergency Medicine, Université de Montréal, 2900, boulevard Édouard-Montpetit, Montreal, Quebec H3T 1J4, Canada; Department of Social Work, Research Center on Aging, Université de Sherbrooke, 3001, Sherbrooke, Quebec J1H 5N4, Canada; Department of Family Medicine, Université de Sherbrooke, 3001, 12e Avenue Nord, Sherbrooke, Quebec, J1H 5N4, Canada; Department of Health Management Evaluation and Policy, School of Public Health, Université de Montréal, 7101 Park Avenue, Montreal, Quebec H3N 1X9, Canada; Research Centre of the Centre Hospitalier de l’Université de Montréal (CRCHUM), 900 rue Saint‑Denis, Montreal, Quebec H2X 0A9, Canada

**Keywords:** causal mediation analysis, mediation analysis, primary care, primary care physician, decision-making, policy makers, policy, family physician

## Abstract

**Background:**

Causal mediation, a quantitative analysis method, has the potential to be a valuable addition to any primary care provider, researcher, or student’s toolbox.

**Objective:**

This manuscript describes the theory behind causal mediation, provides a running example to help understand the application of this method in research, and explains how the results may be applied practically to help design appropriate interventions.

**Methods and application:**

Causal mediation allows an exploration of the mechanism of action of a primary care intervention on an outcome that may pass through a third variable that is on the causal pathway, a mediator. Causal mediation analysis allows the decomposition of the total effect of an intervention on an outcome into both direct and indirect effects. Careful interpretation of generated results can guide decision-makers when devising or refining interventions or policies that affect patient health outcomes in primary care.

**Conclusion:**

Causal mediation has been used in many disciplines and is well-positioned to answer varied research questions. However, the full extent of its potential has yet to be realized.

**Key messages**

- Causal mediation overcomes some limitations of traditional mediation.
- Causal mediation decomposes the total effect of an intervention on an outcome.
- Primary care can benefit from the evidence that causal mediation provides.

## Introduction

Causal mediation is a method for better understanding how an intervention influences a mediator, an intermediate variable on the causal pathway between the intervention and the outcome, which in turn impacts an outcome [1, 2]. This quantitative method is finding its way into different disciplines including clinical research [3], medicine [4], education [5], epidemiology [1], psychology [1], and public health [5]. The rising popularity of causal mediation is due in large part to its ability to elucidate the mechanism of the effect of an intervention on an outcome [1]. Results of causal mediation analysis can help policymakers and practitioners target strategies to intervene on the exposure or the mediator(s) depending on which has the most impact on the outcome [1]. Causal mediation therefore has the potential to be a valuable tool in the primary care researcher’s toolbox. However, nontechnical explanations of this analytical method and guidance on interpreting its results are limited. The purpose of this manuscript is to introduce primary care providers, researchers, and students to causal mediation analysis, so as to provide a clear understanding of this method, the interpretation of the results it generates, and its relevance for informing practice and policy decision-making.

## What is a mediator?

**Figure 1.**
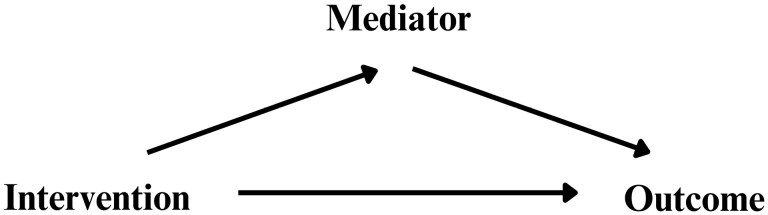
Conceptual map showing the intervention, mediator, and outcome.

When studying the effect of an intervention on an outcome, curiosity and closer examination of the variables may lead to questions about the presence of a third variable on the causal pathway between the intervention (or treatment or exposure), and the outcome [1]. This third variable, a mediator, may exert a partial or total effect on the outcome of interest [6] (Fig. 1).

## Why causal mediation?

The study of mediators is not a recent one, but gained popularity owing to a paper by Baron and Kenny (1986) outlining steps to assess mediation [1, 7]. Later works recommended the “product method” or the “differences method,” often referred to as traditional mediation methods [8]. However, the use of traditional mediation methods is limited and prone to bias [5]. While traditional mediation is still prevalent in the literature, causal mediation has been gaining in popularity since the early 2000s as an alternative method for assessing mediation [8]. Causal mediation analysis overcomes the limitations of traditional mediation methods and allows for models with noncontinuous mediators or noncontinuous outcomes, as well as potential intervention–mediator interaction [8]. Additionally, causal mediation analysis is grounded in a robust causal inference framework and its underlying assumptions that are explicitly stated and evaluated for a causal interpretation [9].

## A working example

To better understand what causal mediation is, consider this hypothetical example. A primary care provider or researcher may be interested in examining the effect of having a regular source of primary care on complications due to poorly managed diabetes. The researcher might hypothesize that since having a regular source of care may improve timely access to primary care, and timely access may in turn help to reduce the number of complications due to diabetes [10], then timely access may be a mediator of the effect of having a regular source of care on the number of complications due to diabetes. In other words, the researcher might pose the following research question:

*Does timely access to primary care mediate the effect of having a regular source of care on the number of complications due to poorly managed diabetes in diabetic patients?*

We can define the intervention, mediator, and outcome in our example as follows:

1. The **intervention** is having a regular source of primary care, *compared* to lacking a regular source of primary care for diabetic patients. This may manifest as reliance on emergency rooms for nonurgent care or visiting more than one healthcare provider.
2. The **mediator** is timely access to primary care, with access envisioned as waiting time for an appointment in the case of an urgent, yet minor health problem. Timely access could have one of two values: good access if a patient can get an appointment with their family physician or another health provider in 2 days or less for an urgent, yet minor health problem (e.g. urinary infection), and bad access otherwise.
3. The **outcome** is the number of times a patient gets complications linked to poorly managed diabetes in the year following measurement of the mediator (e.g. hypoglycemia or hyperglycemia [11]).

## Causal mediation estimates

**Figure 2.**
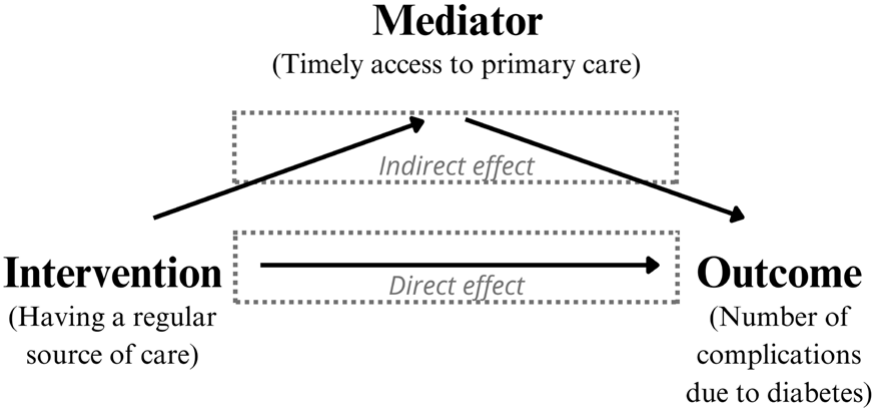
Conceptual map with arrows indicating the direct effect of the intervention on the outcome, as well as the indirect effect through the mediator.

Overall, causal mediation allows the decomposition of the total effect of an intervention on an outcome into a direct and indirect effect known as the natural direct effect, and the natural indirect effect (Fig. 2):

1. The total effect represents the average change in the outcome, had the entire target population received versus did not receive the intervention of interest [12]. This is similar to finding the total effect of an intervention in a randomized controlled trial by subtracting the effect of those in the treatment group from those in the control group. This effect is typically estimated by comparing the outcome in those who received the intervention to those who did not.
2. The natural direct effect, may be considered as the effect of the intervention on the outcome through one or more pathways not involving the mediator under examination [1].
3. The natural indirect effect, can be understood as the effect of the intervention on the outcome that passes through the mediator [1].

It should be noted that the total effect can be further decomposed into four components: the effect due to interaction (reference interaction), the effect due to mediation alone (pure indirect effect), the effect due to both mediation and interaction (mediated interaction), and the effect due to neither mediation nor interaction (controlled direct effect) [1]. However, this method is beyond the scope of this paper and has been described in more detail elsewhere [1].

## Interpretation of causal mediation results

To explain how to interpret causal mediation results based on the posed research question, we will consider fictitious results. The results of causal mediation analysis from a study on the impact of having a regular source of care on the number of complications due to poorly managed diabetes, mediated by the ability to access primary care within 2 days for an urgent yet minor health problem, are presented in Table 1. We will assume that the mediator is binary, i.e. good *versus* bad access and that the outcome is a count, i.e. number of times a patient gets complications linked to poorly managed diabetes [2].

**Table 1.** Results of causal mediation analysis of the fictitious example showing the total effect, natural direct, and natural indirect effects.

Effect	Estimates as relative risk	95% Confidence interval
Total effect	−0.144	[−0.154, −0.134]
Natural direct effect	−0.063	[−0.067, −0.059]
Natural indirect effect	−0.081	[−0.087, −0.074]

In this example, the total effect can be interpreted as the average effect of having a regular source of care on the number of complications due to poorly managed diabetes. The total effect in Table 1 indicates that overall, patients who have a regular source of care have a statistically significant 14.4% lower risk of having complications due to diabetes compared to those who do not have a regular source of care. Table 1 shows that this total effect of 14.4% divides into two subcomponents, the 6.3% that is a direct effect and the 8.1% that is an indirect effect (i.e. 14.4% = 6.3% + 8.1%).

The natural direct effect, the effect of having a regular source of care, operating through pathways not involving access to primary care, corresponds to a 6.3% decrease in the risk of complications due to poorly managed diabetes. This result means that part of the impact of having a regular source of care on the number of complications due to poorly managed diabetes operates through one or more mechanisms other than timely access to primary care. These may include continuity, coordination of care, etc.—other potential mediators not addressed in this research question, but that could be examined in future studies.

The natural indirect effect in this fictitious example is the effect of having a regular source of care on complications due to diabetes, which was due to access within 2 days for an urgent, yet minor health problem. The natural indirect effect results in an 8.1% decrease in the risk of complications due to diabetes. In the case of a well-powered study, had the results of the natural indirect effect not been significant, then we could have assumed that the impact of a patient having a regular source of care was truly direct, meaning that the impact of a patient having a regular source of care on the number of complications due to poorly managed diabetes, does not pass through timely access to primary care.

Overall, causal mediation analysis can now be readily conducted by means of software packages developed over the past decade (e.g. SAS, R, SPSS, Stata, and M*plus*) [8]. Valente *et al*. [13] provide detailed information on how to conduct causal mediation analyses using these packages.

## Causal mediation assumptions

**Figure 3.**
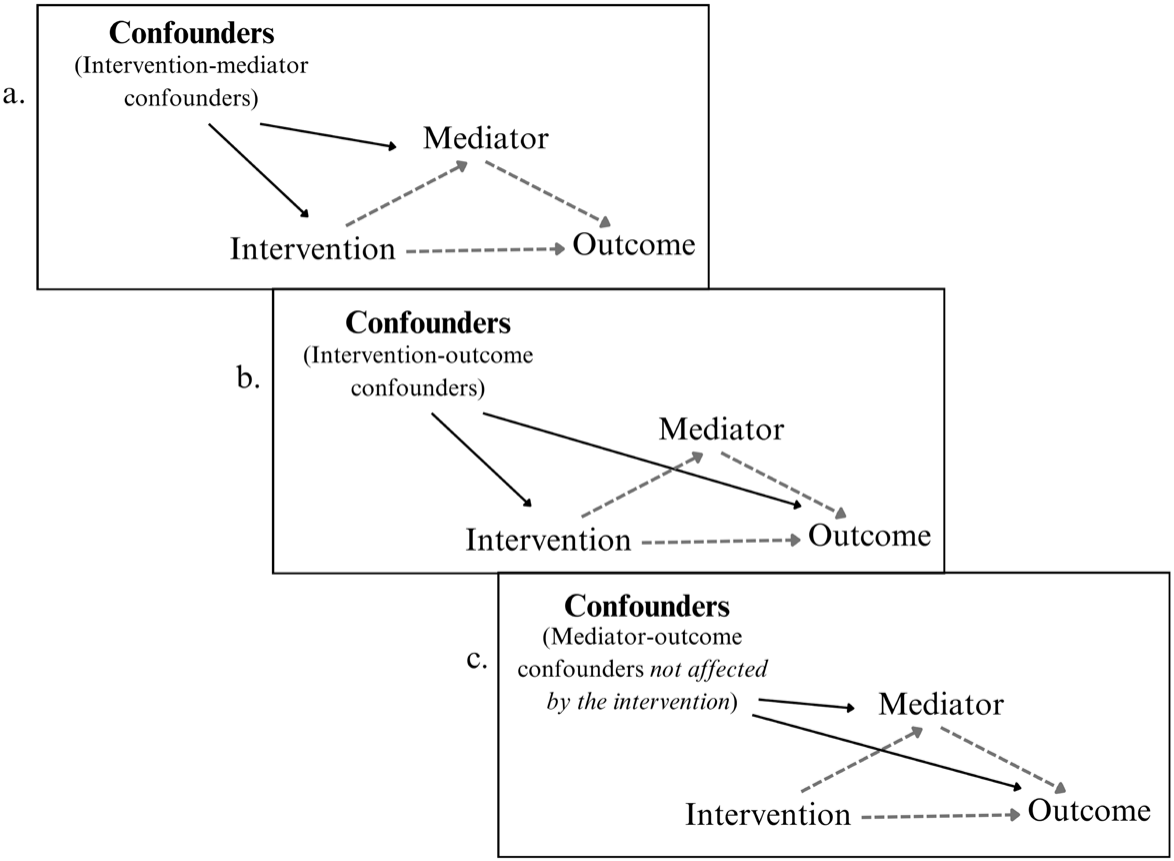
The confounders that should be considered in causal mediation analysis. (a) the intervention–mediator confounders, (b) the intervention–outcome confounders, and (c) the mediator–outcome confounders.

Causal mediation relies on clearly defined assumptions that should be considered for estimates to be interpreted causally [14]. An a priori assumption of causal mediation is temporality [1], which means that the intervention should precede the mediator, which should precede the outcome [1]. One other assumption derived from the causal inference framework is exchangeability [14]. Exchangeability requires that if you interchange the individuals in the intervention group and the control group, the potential effects measured would be the same [2]. This can be achieved by considering and controlling for all measured and unmeasured confounders of the intervention–outcome, mediator–outcome, and intervention–mediator in the analysis (Fig. 3). Moreover, an additional assumption is that no mediator–outcome confounders are themselves affected by the intervention [1]. For an introduction to the causal inference framework and its assumptions within the primary care context, please see Sourial *et al*. [14].

## Insights from causal mediation to inform practice or policy

Researchers may be interested in studying mediation for various reasons. Studying a mediator could help clarify the mechanism of action of the intervention on the outcome, or challenge a proposed theory regarding this mechanism [2]. Studying mediation may be interesting in situations where it is impossible to intervene in an exposure, such as when the exposure is age, or socioeconomic status [1]. In that case, to have an impact on the outcome, it may only be possible to intervene on the mediator, instead of the exposure, when developing targeted interventions [1]. Studying mediation could also serve to identify levers of change to direct practice and policy [2]. In other words, mediation analysis may serve to identify an intervention that is likely to have the greatest impact on the outcome [1].

Results of our fictitious example show that diabetic patients with a regular source of primary care, compared to those without a regular source of primary care, have a lower risk of complications due to poorly managed diabetes. This finding may prompt policymakers to act on timely access to primary care, the mediator, instead of on other quality measures of teams. For instance, new policies may be applied in which health providers prolong their work hours even on the weekends, or provide a hotline that patients can call if they have an emergency.

In some cases, and if we are sure that we have enough power to detect an effect, mediation analysis may reveal that the natural indirect effect is not statistically significant and that most of the effect is direct. In that case, there are two possibilities. Either policymakers may deem it worthy to explore other possible mediators of the effect on the outcome that may be leveraged to impact the outcome. For instance, researchers and policymakers may decide to look into other mechanisms of action of regular providers of primary care, such as their impact on relational continuity, that may potentially decrease the number of complications due to diabetes further. Or, if no other mediators are good candidates for further research, the effect may be truly direct, and then policymakers may focus resources on, e.g. educating diabetic patients about the importance of having a regular source of care.

## Causal mediation in research

Causal mediation can be used to answer different research questions that are important in primary care. For instance, causal mediation may allow a better understanding of how:

- physician job satisfaction may mediate the relationship between patient load and physician burnout.
- relational continuity may mediate the effect of patient-centered medical homes on emergency department use.

Several recent studies have employed causal mediation analysis to answer different research questions in and outside the realm of primary care, or medical research more generally (Table 2).

**Table 2.** Recent research that has used causal mediation methods.

Article	Objective	Exposure, mediator(s), primary outcome	The causal mediation analysis showed that…
(Sugiyama *et al*., 2015) [15]	To investigate the effect of community-based DSME intervention targeting empowerment on mental HRQoL and to determine whether the effect is direct or mediated by glycemic control.	Exposure: diabetes self-management education Mediator: change in HbA1c during the study period as the main mediator of interest Primary outcome: the change in Mental Component Summary score (MCS-12) from the SF-12 Health Survey	the intervention had a direct effect, yet no indirect effects mediated via HbA1c.
(Baker *et al*., 2020) [16]	To examine the association between prenatal acetaminophen exposure measured in meconium and ADHD in children aged 6–7 years, along with the potential for mediation by functional brain connectivity.	Exposure: prenatal acetaminophen Mediator: functional brain connectivity Primary outcome: diagnosis of ADHD	the effect of prenatal acetaminophen on increased child hyperactivity was mediated by increased negative connectivity between frontoparietal and default mode network nodes to clusters in the sensorimotor cortices.
(Joyce *et al*., 2022) [17]	To investigate the effects of physical therapy or yoga versus education on back-related outcomes, and possible mediation by psychological mechanisms.	Exposure: physical therapy or yoga Mediators: changes in pain self-efficacy, fear-avoidance beliefs, depression, anxiety, perceived stress, and sleep quality Primary outcome: changes in back-related pain and disability	when the exposure was physical therapy, the effect passed through the mediator perceived stress to impact disability.
(Jung *et al*., 2023) [18]	To investigate how physician burnout impacts change in work hours, and possible mediation by work engagement.	Exposure: physician burnout and work-hour changes Mediator: work engagement Outcome: work hours	work engagement significantly mediated the relationship between burnout on work-hour reduction.
(Song *et al*., 2017) [19]	To investigate the effect of team dynamics on clinical work satisfaction, and possible mediation by primary care coordination between primary care providers.	Exposure: overall team dynamics Mediator: patient care coordination between primary care providers Outcome: clinical work satisfaction	the relationship between team dynamics and satisfaction for attending clinicians was partially mediated by coordination.

## In conclusion

Causal mediation is a powerful tool for advancing an in-depth scientific understanding of the mechanism of the effect of an intervention on an outcome. Not only does accessing the knowledge gained from causal mediation provide information on the mechanism of effect, but it also enables providers, researchers, and policymakers to devise or refine interventions or policies, ultimately improving outcomes for patients and health systems.
